# Anti-C1-inactivator treatment of glioblastoma

**DOI:** 10.18632/oncotarget.26456

**Published:** 2018-12-21

**Authors:** Karolina Förnvik, Jonatan Ahlstedt, Kurt Osther, Leif G. Salford, Henrietta Nittby Redebrandt

**Affiliations:** ^1^ The Rausing Laboratory, Division of Neurosurgery, Department of Clinical Sciences, Lund University, Lund, Sweden; ^2^ Department of Clinical Chemistry, Skåne University Hospital, Lund, Sweden; ^3^ Department of Neurosurgery, Skåne University Hospital, Lund, Sweden

**Keywords:** complement, C1-inactivator, glioblastoma

## Abstract

**Purpose:**

Glioblastoma multiforme (GBM) or astrocytoma grade IV is the most common type of primary brain tumor in adults. In the present study, we investigate the role of the complement system in the glioblastoma situation in an experimental model, since we have previously been able to show a blockade of this system in the glioblastoma setting.

**Technique and results:**

A GFP-positive glioblastoma cell line was used to induce glioblastomas subcutaneously in rats (n=42). Antibodies against C1-Inactivator (C1-IA) were used to try to re-activate the complement system. We were able to demonstrate an increased survival in rats treated with anti-C1-IA with an intratumoral route, and we could establish the same the results in a second series. Serum analyses revealed decreased levels of IL-1b and GM-CSF in animals 24 days after tumor cell inoculation in the anti-C1-IA group when compared to controls. Immunohistochemistry revealed decreased expression of C1-IA following treatment.

**Interpretation:**

These results are in line with our previous work showing an upregulation of C1-IA, which is able to block the classical complement pathway, in glioblastomas. Treatment with antibodies against C1-IA seems to be beneficial in the glioblastoma situation, and no side effects could be seen in our experiments.

## INTRODUCTION

Glioblastoma multiforme (GBM) or astrocytoma grade IV is the most common type of primary brain tumor in adults. It has a capacity to spread and infiltrate normal surrounding brain tissue which limits the multimodality treatment currently used comprising neurosurgery, chemotherapy and radiotherapy, and makes complete resection virtually impossible [1]. In 2005 Temozolomide was approved by the Food and Drug Administration (FDA) for treating GBM and subsequently Avastin (Bevacizumab) has been approved. Immunotherapy has emerged as an important adjuvant sparking further research into this field. In 2016 Kindy et al. [2] showed that treating mice with a single s.c. injection of a semi-allogeneic vaccine in a mouse model of GBM significantly increases survival, granting the author Orphan Drug Designation for irradiated, semi-allogeneic vaccines to be used in a clinical setting for treatment of GBM.

We have recently described that C1-IA (complement1-inactivator), also denoted C1-inhibitor (C1-inh, C1 esterase inhibitor, serpin family G member 1), is overexpressed in glioblastomas on the gene level, protein level, and on glioblastoma cells from patients as well as and in rat glioma cell lines [3], which introduces inactivation of the complement system as an important factor to be considered in glioblastoma research. The complement system comprises three biochemical pathways; the classical, the alternative, and the lectin induced pathways, and under normal conditions the complement system acts as a functional bridge between the innate and the adaptive immune responses [4]. The classical pathway is initiated by activation of the C1 complex, which irreversibly can be bound to and inhibited by C1-IA, the only known physiological inhibitor of the C1r and C1s proteases [5]. Besides its inhibitor activity in the complement system, C1 IA is also known to inhibit proteases of the fibrinolytic, clotting, and kinin pathways, and, additionally it is the most important physiological inhibitor of plasma kallikrein, fXIa, and fXIIa. C1IA is highly glycosylated, bearing both N- and O-glycans. The N-terminal domain is especially heavily glycosylated. C1 IA is a member of the serpins and its inhibitory activity is located in the C domain.

In the present study the main hypothesis was that we could prolong survival if we treated the animals, inoculated with glioma cells, with antibodies against C1-IA. We also wanted to start the search for possible mechanistic explanations to the effects observed. When applying research using experimental immunotherapy on glioblastoma the choice of model is of vital importance. Several old models have been used to date [6] including xenograft models in immunocompromised animals. However, there is a need to use cells as similar to the human condition as possible, meaning that the cells have not been cultured for decades in laboratories and the animals are not immune deficient. Therefore, we have previously introduced the NS1 rat glioma model, which is our new GFP positive tumor cell line. This model was used in the present study to investigate the effects of anti-C1-IA treatment.

## RESULTS

### Increased survival in rats treated with intratumoral anti-C1-IA

**Figure 1:**
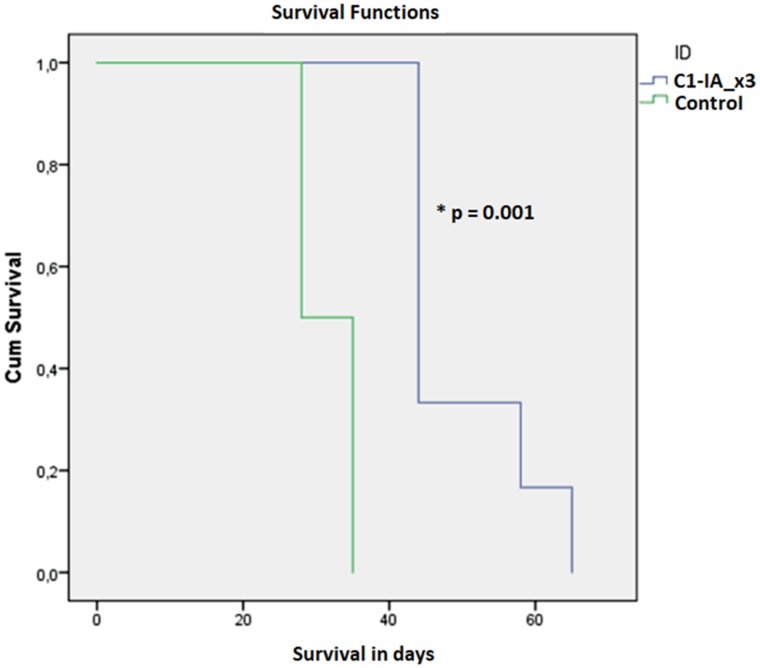
Series 1 Six (6) animals were treated with anti-C1-IA intratumorally days 0, 7 and 14; six (6) animals were untreated control animals. Survival compared across groups with Kaplan–Meier. Survival was significantly increased following anti-C1-IA injections intratumorally (log rank Mantel Cox p = 0.001). Mean survival was 49.83 ± 3.8 days in animals treated with anti-C1-IA (n=6); 31.5 ± 1.6 days in control animals (n=6).

**Figure 2:**
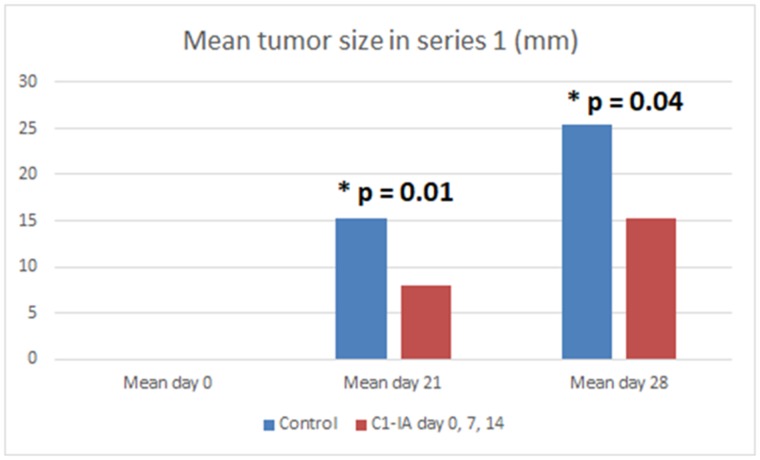
Series 1 Six (6) animals were treated with anti-C1-IA intratumorally on days 0, 7 and 14; 6 animals were untreated control animals. Mean tumor size (mm) in animals with subcutaneous tumors treated with anti-C1-IA intratumorally (days 0, 7, 14 with 3 mg anti-C1IA) and control animals with no treatment at all. There was a significant difference in tumor size between the groups on both day 21 (p = 0.01) and 28 (p = 0.04) as revealed by 2-paired t-test. On day 0 no palpable tumor could be identified.

**Figure 3:**
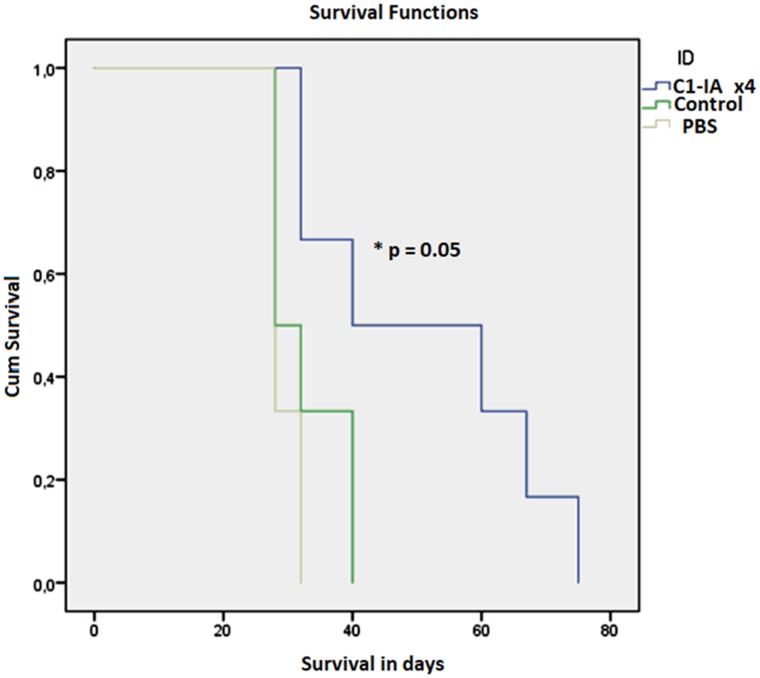
Series 2 Six (6) animals were treated with anti-C1-IA intratumorally on days 0, 7, 14 and 21; six (6) animals were untreated control animals; and 6 animals were treated with PBS intratumorally on days 0, 7, 14, 21. Survival compared across groups with Kaplan–Meier. Survival was significantly increased following anti-C1-IA injections intratumorally (log rank Mantel Cox p = 0.05). Mean survival was 51.0 ± 7.7 days in animals treated with anti-C1-IA (n=6); 32.7 ± 2.4 days in control animals (n=6); and 29.3 ± 0.8 days in animals treated with PBS (n=6).

**Figure 4:**
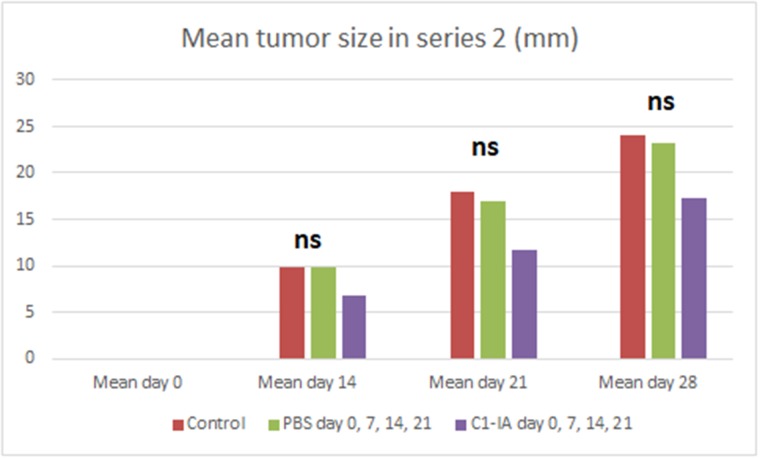
Series 2 Six (6) animals were treated with anti-C1-IA intratumorally on days 0, 7, 14 and 21; six (6) animals were untreated control animals; and 6 animals were treated with PBS intratumorally days 0, 7, 14, 21. Mean tumor size (mm) in animals with subcutaneous tumors treated with anti-C1-IA intratumorally (days 0, 7, 14, 21 with 3 mg anti-C1IA in 0.5ml PBS), control animals with intratumoral PBS (days 0, 7, 14, 21 with 0.5ml PBS) and control animals with no treatment at all. With one-way ANOVA no significant difference in tumor size could be demonstrated between the groups on day 14, 21 or 28 (p > 0.05). On day 0 no palpable tumor could be identified. Survival compared across groups with Kaplan–Meier. Survival was significantly increased following anti-C1-IA injections intratumorally (log rank Mantel Cox p = 0.05). Mean survival was 51.0 ± 7.7 days in animals treated with anti-C1-IA (n=6); 32.7 ± 2.4 days in control animals (n=6); and 29.3 ± 0.8 days in animals treated with PBS (n=6).

Both series of experiments with intratumoral anti-C1-IA treatment enabled us to demonstrate a significant survival advantage upon treatment (Figures 1 and 3). Furthermore, there was a significant decrease in tumor size over time following treatment with anti-C1-IA (Figures 2 and 4). By adding the PBS control in the second series, we could demonstrate that the survival advantage was not due to disruption in the tumor environment of the volume injected or the medium in which the antibody was administered, but rather the findings indicated that the benefits observed, could be attributed to the antibody treatment. We could not identify any side effects of the treatment in regard to the general condition of the animals.

### Immunological effects seen in serum samples

**Figure 5:**
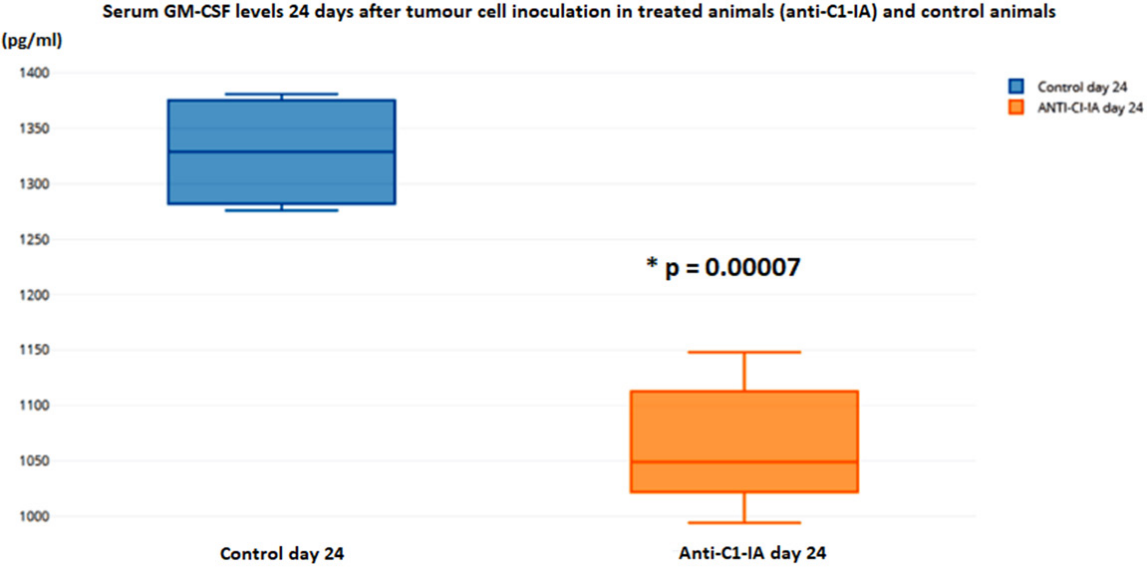
Serum levels of IL-1b were not different in the animals 10 days after tumor cell inoculation, but on day 24 there was a statistically significant decrease in the animals treated with anti-C1-IA intratumorally (units in pg/ml)

**Figure 6:**
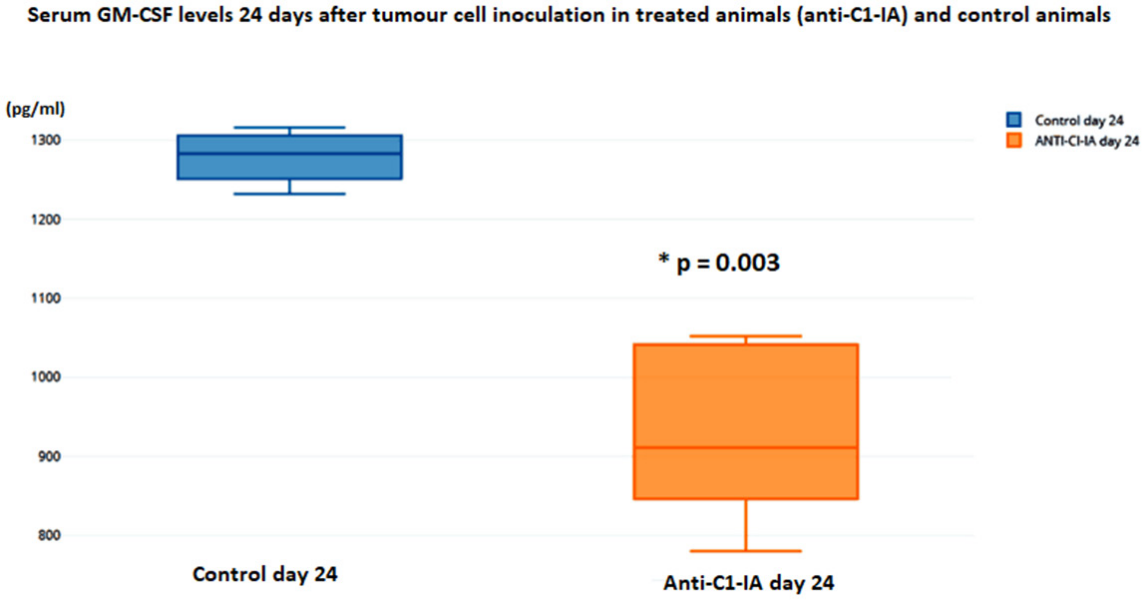
Serum levels of GM-CSF were not different in the animals 10 days after tumor cell inoculation, but on day 24 there was a statistically significant decrease in the animals treated with anti-C1-IA intratumorally (units in pg/ml)

Using the Bioplex technology for immunological responses in serum, no difference between treated animals or control animals was seen 10 days after tumor cell inoculation and after the first dose of antibody treatment. However, 24 days after the tumor cell inoculation, at a time point where the animals had received treatment twice, this technology revealed alterations. In the antibody treated group, there was a significant decrease in the IL-1b expression (t-test p = 0.00007) (Figure 5) and GM-CSF (t-test p = 0.0003) (Figure 6) in serum.

### Immunohistochemical results

**Figure 7:**
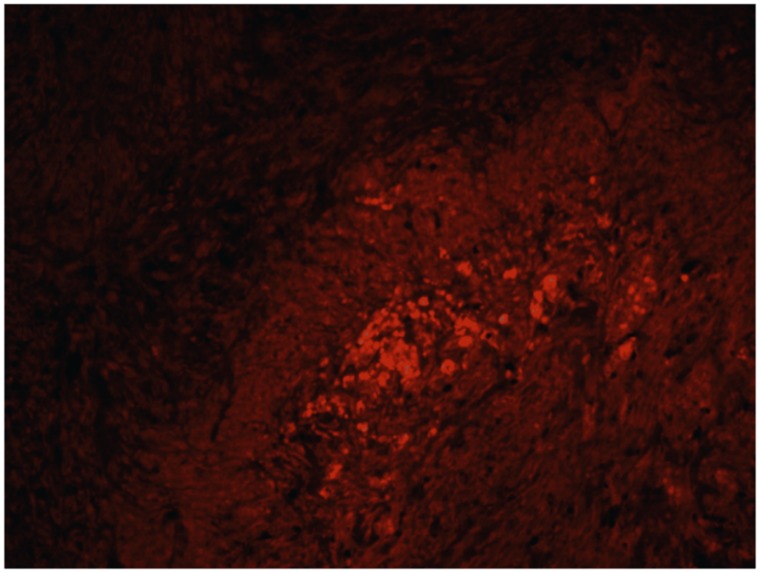

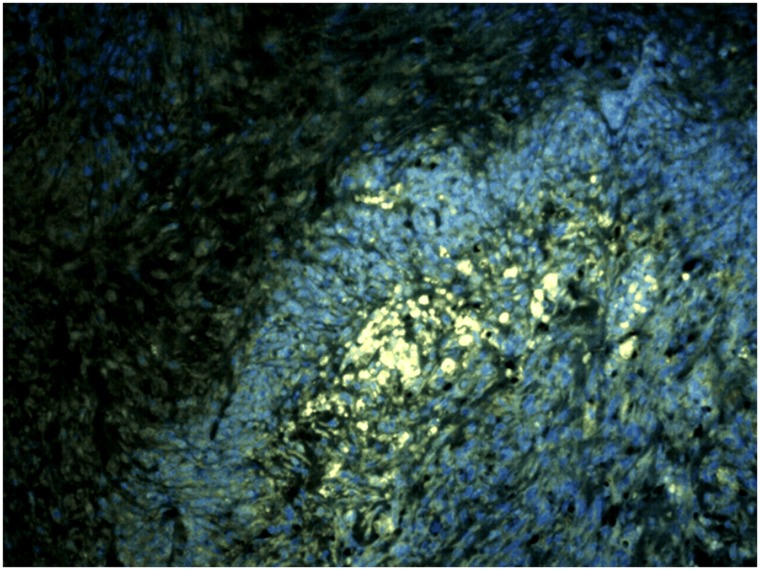
**(A)** and **(B)**. Staining with labelled antibody against C1-IA and with Alexa Fluor 594-conjugated goat anti-rabbit serum in a control (untreated) animal demonstrated that the protein could be detected in the tumor post mortem **(A)**. Intense GFP staining **(B)** was surrounded by nuclei, indicative of a tumor zone co-localized with the anti-C1-IA staining method. However, in an anti C1 IA treated animal, no such signal could be detected.

Using fluorescence microscopy fitted with appropriate wave length filters, we could see that there was no staining for C1-IA protein in a sample section from an animal treated with anti-C1-IA antibody intratumorally. Conversely, in a sample section from a control animal, we found intense GFP signal indicative of the presence of rat NS1 tumor cells; there was also a distinct staining of the cells from the anti-C1-IA antibodies, - actually, the same cells, which showed GFP signal (Figure 7).

## DISCUSSION

In the present study, we could, for the first time to our knowledge, demonstrate that intratumoral injections with anti-C1-IA significantly prolongs survival and reduces tumor volume in animals with glioblastoma. We could also demonstrate effects seen in the circulation upon antibody administration into the tumor after two doses.

This suggests that this targeted antibody treatment decreasing the presence of postulated surplus of C1 IA, e.g., in the microenvironment of the glioblastoma as a possible approach, might potentially benefit glioblastoma sufferers. Notably, no animal displayed any visible side effects. Furthermore, we were able to demonstrate that the effect was not due to the injection caused by the volume of fluid *per se* entered into the tumor, but actually seemed to be attributed to the activity caused by the antibody itself.

Future studies will focus on intratumoral treatment of intracerebral tumors. Since this approach offers potentially more complicating factors, we chose to test our hypothesis with an easily accessible subcutaneous model. Possible confounding factors in an intracranial model could be that it is difficult to detect potential bleeding and infections, and at the same time establish a high degree of probability as to how we could be sure that the injections were entered into the tumor and not somewhere else, as for instance into the CNS ventricular system. In the hypothetical situation of application of anti C1 inactivator in humans an intrathecal route might be considered, where a Rickham reservoir catheter is installed with the tip placed into the ventricle system in the brain. Another possibility could be to place a catheter in the resection cavity in connection to the surgical removal of the glioblastoma. It would of course also be possible to monitor the circulating C1 inactivator in the blood. However, it is important to point out that additional preclinical research is needed before this can be considered. Also, the question of the blood-brain barrier needs to be addressed, and other possible routes for delivery of the antibody into the tumor should be tested experimentally.

It was intriguing to find that the treatment with anti-C1-IA actually could decrease the serum levels of GM-CSF. This might have immunological implications, which could possibly explain some of the positive effects seen by our treatment. It has been suggested that GM-CSF is synthesized by glioblastoma cells themselves, and that this leads to an immunosuppressive shift that generates a relative lymphopenia, which enhances growth and motility of glioblastomas [7]. Kohansbash et al. [8] described that GM-CSF plays a central role for the induction of IL-4Rα expression on myeloid cells, and that GM-CSF is upregulated in both human and mouse glioma microenvironments compared with normal brain or peripheral blood samples. They have suggested a GM-CSF-induced mechanism of immunosuppression in the glioma microenvironment via upregulation of IL-4Rα on MDSCs (Myeloid-derived suppressor cells). However, the role of GM-CSF also seems to be a dual one. Others have reported that GM-CSF secreting immunotherapy in combination with other therapies could reduce the tumor volume [9]; worth noting is that Li et al’s study [9] was based on a “colon model”, and not a glioblastoma model.

In our serum analysis we could also observe a down-regulation of IL-1b after administration of anti-C1-IA. Regarding IL-1b it has been described that this substance is released by glioblastoma cells both *in vitro* and *in vivo* [10]. Furthermore, it has been suggested that IL-1b induces changes affecting the glioma microenvironment in favor of increased tumor invasion, migration and angiogenesis [11]. In other cancer types, such as pancreatic cancer, it has been shown that the possession of a certain genotype results in an increased IL-1b production, which was associated with shortened survival and increased serum CRP level [12].

## MATERIALS AND METHODS

### Cells

The rat glioblastoma cell line NS1, previously described by Nittby et al. [13], was used. NS1 is a new GFP positive tumor cell line which was created by ENU treatment of pregnant homozygous GFP-positive Fischer 344 rats, where the offspring developed GFP-positive CNS-tumors, resulting in the NS1 cell line [13]. Rats inoculated with NS1 cells, either intracranially or subcutaneously, develop cell-rich tumors with an invasive growth pattern, as can easily be tracked since the tumor cells express GFP. The tumors are positive for GFAP and the tumor cells have been shown to have a strong RNA expression for wt IDH1, wt p53, IDO1 and EGFR, as described earlier [13].

In the present study, the rat glioma cells (NS1) were cultured using RPMI-1640 (Sigma-Aldrich) medium with addition of 1% ml Na-pyruvate, 1% ml HEPES (4-(2-hydroxyethyl)-1-piperazineethanesulfonic acid), 0.1% ml gentamycin, as well as 10% inactivated fetal calf serum (heated to 56 °C for 30 minutes).

After culturing in T25 flasks, the cells were prepared for inoculation by removal of the medium and washed gently with PBS. Trypsin, e.g., TrypLe TM Express (Invitrogen) was added and the cells were incubated in 37°C for 1-2 minutes in order to detach the adherent cells from the flask. Additional medium was added and viable cells were counted. The cells were centrifuged at 1200 rpm for 5 minutes at 4°C, then the supernatant was carefully removed to avoid any potentially immunogenic calf serum. Afterwards the cell pellet was re-suspended in serum-free medium (R0) to and adjusted to achieve the concentration used for inoculation, as previously described [3].

### Antibodies

Intratumoral treatment was achieved using rabbit anti-rat C1 inactivator (Covance, USA). The antibody was dissolved in PBS at a concentration of 6 mg/ml. 0.5 ml of antibody solution was used for each treatment and hence the corresponding volume of PBS was used for intratumoral injection of the control animals.

### Animals

Fortytwo (42) female Fischer 344 rats (Fischer Scientific, Germany) were included in the study in three series of experiments. These animals were inoculated with 50 000 NS1 glioblastoma cells subcutaneously on their right hind leg, performed under isofluorane inhalation anaesthesia, as previously described [13].

The rats were housed in pairs with ad libitum access to water and rat chow. The animals were monitored for tumor growth and once the tumor size exceeded 30 mm or penetrated through the skin to cause open wounds the rat was euthanized and the tumor saved for histological analysis.

### Ethics statement

The experiments were approved by the regional ethics board in Lund, Sweden (permit number M102-16). All efforts were made to minimize animal suffering.

#### Series 1

Six (6) rats were treated with intratumoral injection of 3 mg anti-C1 inactivator antibody (Covance, USA) suspended in 0.5 ml phosphate buffered saline (PBS) on days 0, 7 and 14, whereas 6 rats remained as untreated controls. Tumor growth was monitored on a regular basis and tumor size measured and recorded on all animals.

#### Series 2

Six (6) rats were treated with intratumoral injection of 3 mg anti-C1 inactivator antibody (Covance, USA) suspended in PBS on days 0, 7, 14 and 21. Six (6) rats received intratumoral injection of 0.5 ml of PBS on days 0, 7, 14 and 21, whereas 6 animals remained as untreated controls. Tumor growth was monitored on a regular basis and tumor size measured and recorded for all animals.

#### Series 3

In a third series the serum response to intratumoral C1-IA was investigated in 12 rats, each receiving either intratumoral anti-C1 inactivator antibody (Covance, USA) on days 7 and 14 post inoculation (n=6) or no injection (n=6 control animals). Blood serum was analysed on day 10 or 24 using Bio-Plex 200 analysis (Multiplex immunoassays), a Luminex Technology based on flow cytometry immunoassays, performed at BMC Lund, Sweden. Using the Bioplex technique, expressions of IL-1a, IL-1b, IL-2, IL-4, IL-5, IL-6, IL-10, IL-12, IL-13, GM-CSF, IFN-g and TNF-α were analysed. On day 10 after tumor inoculation, serum for analyses was obtained from 4 treated animals and 4 controls. On day 24 after tumor inoculation, serum for analysis was obtained from 5 treated animals and 4 controls.

### Immunohistochemistry

Representative animals were selected for analysis. All the rats used in the experiment were initially inoculated subcutaneously with an amount of 50,000 NS1 rat glioma cells. Tumors isolated from the rats were fixed using Phosphate-buffered 4% Paraformaldehyde as described previously [3], paraffin embedded and sectioned using a microtome. The primary polyclonal antibodies were diluted 1:400 in PBS containing 1% BSA and 2% normal goat serum in the following manner; the fixed tissue samples were incubated with rabbit anti-rat C1 inactivator (Covance, USA), permeabilized with 0.1% Triton^™^ X-100 for 10 minutes, blocked with 1% BSA for 1 hour and labeled with 2 μg/mL the rat primary antibody for 1 hours at room temperature. The tissue was subsequently washed and incubated for 1 hour at room temperature with secondary antibodies consisting of Alexa Fluor 594-conjugated goat anti-rabbit serum (ab150084 Abcam) at a concentration of 0.5μg/mL in phosphate buffered saline containing 0.2% BSA at room temperature. After washing with PBS, the tissue was mounted with anti-fading vecta-shield mounting medium with 4,6-diamidino-2-phenylindole (nuclear stain with DAPI) (Vector Laboratories Inc., Burlingame, USA) and were photographed using fluorescence microscope fitted with the appropriate wavelength filters.

### Statistical analysis

*In vivo* experiments were evaluated using 2-tailed student’s t-test assuming equal variance. Kaplan–Meier and log rank Mantel cox test were used to analyze survival using SPSS.

## CONCLUSIONS

In the present *in vivo* study we were able to continue and expand our previous work on exploring the role of C1-IA in glioblastoma. We could demonstrate that intratumoral treatment with anti-C1-IA resulted in prolonged survival and reduced tumor burden. Furthermore, we have presented possible mechanistic contributions to the treatment effect observed. We believe that this encourages further work into the previously unestablished role of the complement system in glioblastomas.
